# Synergizing Attribute-Guided Latent Space Exploration (AGLSE) with Classical Molecular Simulations to Design Potent Pep-Magnet Peptide Inhibitors to Abrogate SARS-CoV-2 Host Cell Entry

**DOI:** 10.3390/v17060828

**Published:** 2025-06-07

**Authors:** Farhan Ullah, Aobo Xiao, Shahid Ullah, Na Yang, Min Lei, Liang Chen, Sheng Wang

**Affiliations:** 1 Tongji Hospital, Tongji Medical College, Huazhong University of Science and Technology, Wuhan 430030, China; farhan@hust.edu.cn; 2 Key Laboratory of Molecular Biophysics of the Ministry of Education, Huazhong University of Science and Technology, Wuhan 430030, China; leimin@hust.edu.cn; 3 Lab for Computational and Structural Biology, College of Life Science and Technology, Huazhong University of Science and Technology, Wuhan 430030, China; 4 School of Artificial Intelligence & Automation, Huazhong University of Science and Technology, Wuhan 430074, China; albert_shaw@hust.edu.cn; 5 S-Khan Lab Takht Bhai, Takht-i-Bahi 55100, Pakistan; drskbioch@gmail.com; 6 State Key Laboratory of Medicinal Chemical Biology, Nankai University, Tianjin 300071, China; yangnanku@nankai.edu.cn; 7 Urology Department, Tongji Hospital, Tongji Medical College, Huazhong University of Science and Technology, Wuhan 430030, China

**Keywords:** deep learning, variational autoencoders (VAE), Wasserstein autoencoders (WAE), SARS-CoV-2, molecular docking, molecular dynamics simulation, Omicron variant

## Abstract

The COVID-19 infection, caused by severe acute respiratory syndrome coronavirus 2 (SARS-CoV-2), has evoked a worldwide pandemic. Even though vaccines have been developed on an enormous scale, but due to regular mutations in the viral gene and the emergence of new strains could pose a more significant problem for the population. Therefore, new treatments are always necessary to combat future pandemics. Utilizing an antiviral peptide as a model biomolecule, we trained a generative deep learning algorithm on a database of known antiviral peptides to design novel peptide sequences with antiviral activity. Using artificial intelligence (AI), specifically variational autoencoders (VAE) and Wasserstein autoencoders (WAE), we were able to generate a latent space plot that can be surveyed for peptides with known properties and interpolated across a predictive vector between two defined points to identify novel peptides that exhibit dose-responsive antiviral activity. Two hundred peptide sequences were generated from the trained latent space and the top peptides were subjected to a molecular docking study. The docking analysis revealed that the top four peptides (MSK-1, MSK-2, MSK-3, and MSK-4) exhibited the strongest binding affinity, with docking scores of −106.4, −126.2, −125.7, and −127.8, respectively. Molecular dynamics simulations lasting 500 ns were performed to assess their stability and binding interactions. Further analyses, including MMGBSA, RMSD, RMSF, and hydrogen bond analysis, confirmed the stability and strong binding interactions of the peptide–protein complexes, suggesting that MSK-4 is a promising therapeutic agent for further development. We believe that the peptides generated through AI and MD simulations in the current study could be potential inhibitors in natural systems that can be utilized in designing therapeutic strategies against SARS-CoV-2.

## 1. Introduction

In late 2019, the SARS-CoV-2 outbreak, also known as Severe Acute Respiratory Syndrome Coronavirus 2, caused a respiratory illness known as COVID-19, resulting in significant damage to worldwide public health [1]. The first cases were reported in Asian countries and spread worldwide [2]. The World Health Organization first reported the illness on 31 December 2019 and declared a global pandemic on 11 March 2020. To date, 7,003,732 people have died from the COVID-19 pandemic. The rapid spread of the virus required the development of safe and efficient control and treatment strategies against its causative agent. Peptides with therapeutic potential have been increasingly studied over the past few decades, resulting in a growing number of FDA-approved peptide drugs [3]. They offer numerous advantages, like ease of synthesis and high specificity. Furthermore, peptides are a satisfactory agent in developing medications and vaccines against infectious diseases, including HIV, herpes, dengue virus, chronic hepatitis, influenza, and SARS-CoV-19 [4]. Antiviral peptides (AVPs) inhibit a virus’s life cycle by interacting with the host or virus. Several antiviral peptides are available naturally in the universe, while others are designed using different computational approaches, such as in-silico mutagenesis and machine learning [5].

Since the virus emerged, the scientific community has been in a race to design effective medicines and a vaccines to treat COVID-19 infection [6]. Although numerous antiviral drugs and vaccines have been developed, the rapid mutation and appearance of novel strains like Delta and Omicron have highlighted the need to develop new treatments [7]. These new variants motivate researchers to utilize innovative approaches for designing novel inhibitors, including peptide-based inhibitors with significant potential such as specificity, adaptability, and efficacy in targeting viral proteins [8]. The computational design of peptides using artificial intelligence to investigate amino acid sequence spaces has become a prominent technique in drug discovery.

Despite advancements in computational resources, designing peptide inhibitors remains a substantial challenge. Two main types of computational methods, structure-based and sequence-based, have been developed for this purpose [9,10]. Structure-based drug design is a significant approach in rational drug discovery, which is designed from a protein pocket or an existing peptide motif bound to the protein [11]. Computational work on the structure-based peptide design involves tasks such as peptide structure prediction, molecular docking, and calculating binding energies [12].

Furthermore, recurrent neural network (RNN)-based variational autoencoder (VAE) models have emerged as an advanced method for sequence-based analysis and represent a cutting-edge approach to peptide design. VAE is well-known for automatic text processing and generation [12] and has been successful in modeling the latent space of sequential data [13]. Previously, several researchers have designed peptide and small-molecule inhibitors. For example, Lijuan Yang reported anticancer peptide inhibitors targeting breast and lung cancer utilizing the Wasserstein autoencoder model VAE [14]. Ellen Van Damme and coworkers identified JNJ-9676, a small-molecule inhibitor targeting the coronavirus M protein [15]. Similarly, Shun Nakamura designed a mutation-tolerant peptide inhibitor against SARS-CoV-2 [16].

In this study, a variational autoencoder (VAE) and a Wasserstein autoencoder (WAE) with structure-based modeling and molecular dynamics simulation were utilized to efficiently generate high-affinity peptide inhibitors targeting the RBD of the SARS-CoV-2 Omicron variant [17]. This model provides an efficient way of predicting potential antiviral peptides, allowing us to explore and generate new peptide sequences with specific properties. After the generation phase, new peptides were constructed using Alphafold 3.0, and molecular docking was performed using the HADDOCK server to make the complexes. Subsequently, the top four complexes out of fifteen were subjected to 500 ns molecular dynamics simulations to evaluate their stability and interaction pattern with the RBD of SARS-CoV-2. The molecular dynamics simulation showed that the new peptides maintain a stable interaction with the RBD. Furthermore, we evaluated the inhibitory potential of the peptides via comprehensive molecular dynamics analyses, including RMSD, RMSF, hydrogen bond analysis, principal components analysis, solvent-accessible surface area, dynamic cross-correlation, and binding free energy calculation (e.g., MM/GBSA).

## 2. Materials and Methods

Despite significant advancements in antiviral drug design, traditional approaches often face limitations in exploring large conformational spaces and optimizing multifunctional properties simultaneously. Deep generative models were utilized, particularly variational autoencoders (VAE) and Wasserstein autoencoders (WAE), to design antiviral peptides for treating COVID-19 infection [17,18]. Generative models offer an automated method for designing new peptides with strong antiviral properties, including high binding affinity and minimal toxicity, distinguishing them from traditional approaches [19]. Due to their capacity to navigate extensive conformation spaces within structural and functional constraints, generative models demonstrate considerable potential in molecular design.

### 2.1. Model Selection and Comparative Analysis

The choice of the VAE/WAE framework for predicting antiviral peptides was driven by its unique strengths compared to alternative generative models, such as GANs and transformers. Below is a concise comparative analysis of these models [20]:

Training Stability: VAE/WAE provides more stable training compared to GANs, which often suffer from issues like mode collapse and vanishing gradients. This stability ensures consistent performance during the generation of novel peptide sequences.

Latent Space Structure: VAE/WAE inherently structures the latent space through regularization techniques (e.g., KL divergence and MMD), enabling meaningful interpolation and structured generation of peptide sequences. This structured latent space is crucial for tasks like property optimization and constrained generation.

Computation and Structure: Models using recurrent architectures (e.g., VAE/WAE) like GRUs are computationally efficient for processing sequential data. This efficiency is beneficial when dealing with large datasets of peptide sequences.

Several studies have demonstrated the effectiveness of VAEs and WAEs in sequence generation tasks. For example, VAEs have been successfully applied in generating protein sequences with desired properties [21]. Similarly, WAEs have shown promising results in generating structured data while maintaining a balance between sample quality and diversity [22].

In contrast, while GANs excel in generating high-quality samples for image data, their application to sequence data remains challenging due to training instability and difficulties in evaluating generated sequences. Transformers, while powerful for sequence modeling, require significant computational resources and lack the inherent probabilistic framework that VAE/WAEs provide [23].

#### 2.1.1. Dataset Generation from Structural Insights

A specialized dataset based on known antiviral peptides was developed to establish a robust training foundation. The dataset construction process was guided by the observation that the peptide adopts a magnet topology characterized by two adjacent α-helices, inspired by the scaffold of PDB ID 7DTL. This structural motif, hereafter referred to as a ‘magnetic-shape structure’ because of its paired helical arrangement, demonstrates enhanced target binding affinity compared to single peptides. The observed improvement is consistent with prior studies revealing that multi-helix peptides, such as double- or triple-helix structures, show stronger molecular interactions due to increased avidity and complementary surface contacts [24].

Data augmentation techniques were employed to enhance dataset diversity, including random mutations, synthetic peptide sequences, and in silico modeling. These methods ensured that the generated peptides explored a wider range of conformations while preserving biologically relevant properties. These structures provided a broad conformational space for peptide generation while maintaining the necessary structural specificity for antiviral activity. Among them, peptides with partial magnet-shaped alpha-helix structures from PDB ID 7DTL were initially selected as the primary scaffolds. Subsequently, selective mutations were introduced on outward-facing amino acid residues to enhance binding affinity, reduce toxicity, and improve structural stability [25].

**Figure 1:**
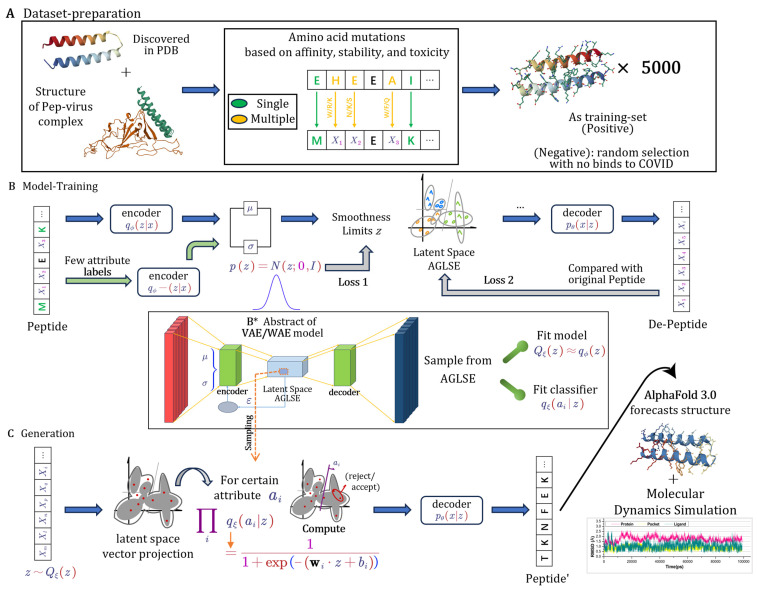
The model stages of antiviral-pep generation with attribute guidance. (**A**) Applying amino acid mutations based on affinity, stability, and toxicity to protein structures from PDB to create peptide datasets. (**B**) Training VAE/WAE model to map peptide sequences into latent space with attribute-guided exploration. (B*) A simplified abstract of the VAE/WAE model. (**C**) Sampling the latent space to generate peptides with specific attributes, followed by structure prediction using AlphaFold 3.0 and molecular dynamics simulations.

As a result, a dataset comprising approximately 5000 unique peptide sequences was constructed, each retaining the overall structural integrity while introducing variations at key functional sites. The dataset generation process, which involved residue mutations for affinity, stability, and toxicity, is illustrated in Figure 1A. This diverse dataset was then used to train the VAE/WAE model, enabling it to learn a rich latent representation of peptides and improving its ability to generate novel antiviral candidates [26].

#### 2.1.2. Core Components and Regularization of Model

The VAE framework is composed of two main components: an encoder $q_{\varphi }\left(z|x\right)$, which maps input sequences to a latent representation z, and a decoder $p_{\theta }(x|z)$, which reconstructs the sequence from this latent space. The primary objective is to optimize the following equation to maximize the evidence lower bound (ELBO):

(1) $$L(\theta ,\varphi ;x)=E_{q_{\varphi }(z|x)}\left[logp_{\theta }(x|z)\right]-D_{KL}\left(q_{\varphi }(z|x)∥p(z)\right)$$

In this equation

x: The input data, represents the peptide sequences.

z: The latent variable capturing the encoded representation of x.

$q_{\varphi }(z|x)$: The encoder function that approximates the posterior distribution of z given x.

$p_{\theta }(x|z)$: The decoder function that reconstructs x from z.

p(z): The prior distribution over the latent variable z is typically chosen as a standard normal distribution N(0,I)

$E_{q_{\varphi }(z|x)}[logp_{\theta }(x|z)]$: The reconstruction loss measures how well the decoder can reconstruct the input data.

$D_{KL}(q_{\varphi }(z|x)∥p(z))$ parallel p(z)): The Kullback–Leibler (KL) divergence ensures that the learned latent space approximates the prior distribution p(z).

This framework is particularly suitable for generating peptide sequences, as it effectively models complex data distributions, making it highly useful for antiviral peptide design. The VAE/WAE model training procedure involved mapping peptide sequences into a latent space with the guidance of specific attributes, as shown in Figure 1B.

One challenge with VAEs is the potential for posterior collapse, where the latent space fails to capture the variability of the input data adequately. To address this issue, the Wasserstein autoencoder (WAE) employs a regularization method that penalizes the divergence between the latent distribution and the prior using maximum mean discrepancy (MMD), expressed by the following equation:

$$L_{WAE}=E_{q_{\varphi }(z|x)}\left[logp_{\theta }(x|z)\right]+\lambda \cdot MMD(q_{\varphi }(z),p(z))$$

where $MMD(q_{\varphi }(z),p(z))$ represents the maximum mean discrepancy, which measures the difference between the latent distribution $q_{\varphi }(z)$ and the prior *p*(*z*).

α: is a hyperparameter that balances the trade-off between reconstruction accuracy and the smoothness of the latent space.

This approach improves the diversity of the generated protein sequences, which is critical in biological applications where diverse functional properties are often needed, particularly when designing proteins capable of inhibiting viral interactions.

#### 2.1.3. Attribute-Guided Latent Space Exploration (AGLSE)

AGLSE, a machine learning model, was proposed to generate new peptides with specific antiviral properties. This method leverages classifiers to introduce attribute-specific guidance within the latent space of the VAE/WAE model, enabling the targeted generation of protein sequences with predefined characteristics such as viral entry inhibition or replication prevention [27]. Each attribute classifier is trained to predict the presence of a specific property based on the latent vector z, and the latent space is explored with the guidance of these classifiers to ensure the generated proteins exhibit the desired properties.

Given a set of attributes $A=(a_{1},a_{2},\ldots ,a_{n})$ representing specific protein properties, the latent space is explored by sampling the conditional distribution as follows:

$$p(z|A)=p(z)\prod_{i=1}^{n}p(a_{i}|z)$$

where $p(a_{i}|z)$ represents the likelihood of an attribute $a_{i}$ given the latent vector *z*. This method steers the generation process toward sequences that are highly probable to possess the desired antiviral properties. The generation of protein candidates, leveraging attribute-guided latent space exploration to ensure the desired antiviral properties, is elaborated in Figure 1C.

Unlike traditional sampling techniques, AGLSE efficiently generates peptide candidates by exploiting the latent space structure while ensuring multiple predefined characteristics, such as binding affinity, stability, and low toxicity. Furthermore, this approach offers greater control over the generation process by manipulating the latent variables, making it an effective strategy for antiviral peptide design with the desired functionalities [28,29].

#### 2.1.4. Training and Optimization

The VAE/WAE model was trained using a custom dataset for antiviral peptide generation. The dataset comprises approximately 5000 peptide sequences, including naturally occurring and synthetically modified sequences designed to enhance antiviral properties, particularly those targeting the SARS-CoV-2 virus. Sequences with lengths between 50 and 80 residues were chosen to improve model generalization, as this range is typical for antiviral proteins.

During the training process, the reconstruction loss was minimized, and a divergence penalty was used to ensure a well-structured latent space. The model architecture consisted of a GRU-based encoder–decoder network with gating mechanisms, mapping input sequences into a 100-dimensional latent space. Depending on the configuration, either a standard normal prior N (0, I) or a Wasserstein autoencoder (WAE) with maximum mean discrepancy (MMD) regularization was used to structure the latent space, ensuring meaningful interpolation and structured peptide generation. The training was conducted with a batch size of 64 using the Adam optimizer (epoch = 250, initial learning rate 0.001, β_1_ = 0.9, β_2_ = 0.999), and gradient clipping (norm threshold = 5) was applied to stabilize updates. Dropout (rate up to 0.3) and weight regularization were incorporated to mitigate overfitting and improve generalization to unseen protein sequences. Additionally, maximum mean discrepancy (MMD) regularization was applied in the WAE model to ensure latent space smoothness, which facilitates controlled sampling [17].

The VAE/WAE model was constructed using the PyTorch framework (version 1.7.1) [30], leveraging its dynamic computation graph for flexible orchestration of the autoencoder architecture and efficient implementation of training processes, including Adam optimization, automatic differentiation, and gradient clipping. The generated peptides were assessed based on their physicochemical properties, including residue characteristics such as polarity, H-bond donor/acceptor, aromaticity, hydrophobicity, and binding affinity, as these are key indicators of antiviral efficacy [31]. These properties were computed using standard bioinformatics tools integrated into the training pipeline.

#### 2.1.5. Peptide Generation

A total of 200 new peptide sequences were generated from the trained latent space, and their structures were predicted using the AlphaFold 3.0 Server [32]. Among these, 15 peptides exhibiting magnet-shaped alpha-helix structures were selected for molecular docking studies. Subsequently, the peptides with the highest binding affinity were subjected to molecular dynamics simulations to assess their structural stability and interaction dynamics with viral targets.

#### 2.1.6. Structure Preparation

The surface area of RBD is large; therefore, the magnet-shaped alpha-helix template PDB ID 7Dtl was used as a template for this study. The crystal structure of the SARS-CoV-2 omicron variant PDB ID 7XAZ [33] was retrieved from the protein databank. Peptides were constructed from the generated sequence via Alphafold 3.0 and named MSK-1, MSK-2, MSK-3, and MSK-4. The SARS-CoV-2 protein was optimized and cleaned by removing the extra ligands and water. Subsequently, energy minimization was performed using the steepest descent approach with 100 steps (step size 0.02 Å) and the conjugate gradient method with 10 steps (step size 0.02 Å) via UCSF Chimera [34].

#### 2.1.7. Molecular Docking

Molecular docking of the SARS-CoV-2 omicron variant and peptides was performed using the high ambiguity-driven protein–protein docking (HADDOCK) algorithm, which utilizes biochemical and biophysical data [35]. The protein and peptides were uploaded to the HADDOCK server in PDB format. Subsequently, we selected the interface residues of RBD and processed the docking analysis. HADDOCK version 2.4 predicted more than 20 complexes with different poses; finally, the best binding pose was selected for further investigation.

#### 2.1.8. Molecular Dynamics Simulation

After the docking process, the complexes were subjected to molecular dynamics simulation to analyze stability, structural dynamics, conformational alterations, and interactions between proteins and ligands [36]. For MD simulation, the Amber22 suite was employed [37,38]. The aim was to explore the dynamic stability of four peptide complexes using the ff14SB force field [39]. Each complex was solvated inside an octahedral box with a minimum distance of 1.0 nm from the border [40]. To imitate the physiological environment and make a charge-free ensemble, the simulation box was neutralized by adding Cl- or Na+ ions with the leap module of the Amber suite [41]. Using the energy minimization protocol, the clashes among residues of the target were fixed. The steepest descent algorithm and conjugate gradient algorithm were utilized for 6000 and 3000 cycles of minimization, respectively [42,43]. After heating up to 300 K, the system was equilibrated at constant pressure (1 atm) using NPT protocol. The Langevin thermostat was used to maintain a constant temperature. Finally, a 500 ns production simulation was performed for each complex through PMEMD CUDA on a supercomputer [44]. Using the particle mesh Ewald approach, long-electrostatic interaction was calculated with a cutoff of 10.0 Å [40]. For covalent bond interaction, the shake algorithm was utilized to fix the hydrogen bond [45]. The cpptraj module of Amber22 was utilized to process the trajectory file from the production simulation. We used Origin v2024 and PyMol [46,47] for data visualization and graphical representation.

#### 2.1.9. Dynamic Cross-Correlation Map (DCCM)

Dynamic cross-correlation motion matrix analysis (DCCM) is a computational technique mostly utilized in molecular dynamics simulations to analyze the correlated motions of residues in biomolecular systems [48]. Specifically, these analyses provide insight into the collective motion and functional conformational changes in protein–peptide complexes and reveal the relative moment of various molecules with each other over the simulation [49]. Furthermore, the DCCM analysis yields a matrix in which each element signifies the correlation of motion between two residues or atoms, with the value ranging from 1 (completely correlated) to −1 (completely anti-correlated). Positive correlation represents the regions moving in the same direction and vice versa. This technique is particularly beneficial to study protein–peptide interaction, conformational motion, and inhibitory activity [50]. DCCM analysis works based on the following equation.

$$C_{ij}=\frac{\langle \left(∆r_{i}\cdot ∆r_{j}\right)\rangle }{\sqrt{\langle {\left(∆r_{i}\right)}^{2}\rangle \langle {\left(∆r_{j}\right)}^{2}\rangle }}$$

In the above equation, *C_ij_* represents the cross-correlation coefficient between atom/residue *i* and atom/residue *j*. Where Δ*r_i_* and Δ*r_j_* are the displacement vectors of atom/residue *i* and *j* from their average positions, and the square root term in the denominator normalizes the correlation coefficient to ensure it ranges from −1 to 1.

#### 2.1.10. Principal Components Analysis PCA

Principal components analysis is a crucial statistical approach commonly used in drug design to analyze and interpret complex multidimensional datasets [51]. PCA provides a solution by decreasing the dimensionality of large datasets while retaining the most significant information, facilitating the discovery of essential factors and patterns important for drug designing [52]. Recent drug design needs extensive data generation, including chemical, biological, and pharmacological information derived from high-throughput molecular docking and other biological experiments [53]. These datasets are frequently multidimensional, comprising several variables such as pharmacokinetic properties, molecular descriptors, and biological activities.

PCA analysis was performed to analyze the significant amount of movement and conformational changes observed in the molecular dynamics (MD) trajectory. By utilizing the cpptraj module of Amber22, the covariance matrix was first calculated by taking the C∞ coordinates and then diagonalized to make eigenvectors and eigenvalues [54]. The concept of eigenvalue refers to the average square fluctuation in the direction of the principal mode.

#### 2.1.11. Binding Free Energy Calculation (BFE)

Binding free energy calculation is a computational approach frequently used to compute the stability and strength of interaction between a ligand and target protein [55]. It describes the energy variation that occurs when ligands bind to the target protein and form a stable complex under equilibrium conditions. Mathematically, the binding free energy (Δ*G*bind) is defined as the difference between the free energy of the complex (ligand–protein) and the sum of the free energies of the unbound ligand and protein in their respective solvated states. MMGBSA.PY script was applied to compute the binding free energy of peptide–protein complexes [56]. The BFE of peptide–protein complexes were calculated, utilizing the last 10 ns MD trajectory, taking 500 snapshots based on the following equation:

Δ*G*_bind_ = Δ*E*_MM_ + Δ*G*_GB_ + γ·SASA − *T*Δ*S*_conf_,

where Δ*E*_MM_ comprises Van der Waals, electrostatic, and internal energy terms. The solvation free energy (ΔGsolv) combines generalized born (GB) electrostatics and a nonpolar SASA.

## 3. Results

### 3.1. Interface Analysis and Mechanism of Viral Interaction

**Figure 2:**
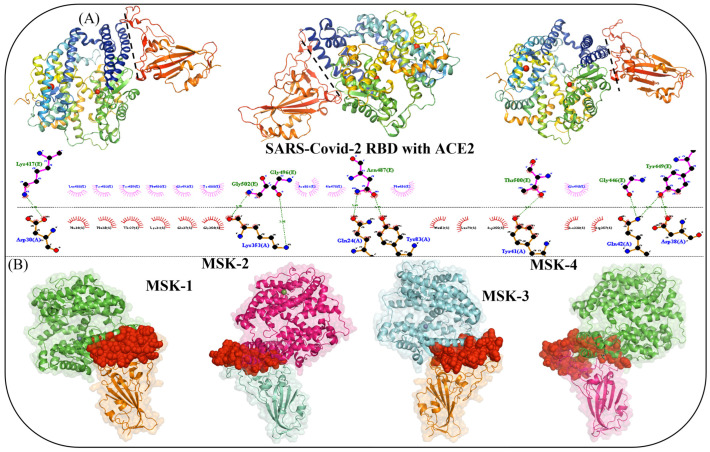
(**A**) The binding poses and LigPlot analysis of RBD and human ACE2 protein (**B**). Molecular docking poses of the constructed peptides with RBD.

The molecular mechanism of SARS-CoV-2 entry remains a critical area in understanding how to block its infection. Coronavirus enters the host cells in three different ways: the first is receptor-mediated endocytosis, receptor-mediated plasma membrane fusion, or antibody-dependent viral entry. The receptor proteins present on the surface of host cells are vital for viral attachment for both endocytosis and fusion. Further investigations into the interaction between SARS-CoV-2 and its receptors have provided an understanding of virus transmission and have led to a solid foundation for the discovery of novel strategies for treatment. The interaction between the SARS-CoV-2 Omicron variant RBD and ACE2 was analyzed using LigPlot software v.2.2 [57], and their binding pose was visualized manually with PyMol v.2.6. The LigPlot analysis revealed that seven residues, including Lys417, Gly502, Gly496, Asn487, Thr500, Gly446, and Tyr449, located on the surface of the RBD of the Omicron variant, are critical for binding interactions with human ACE2, as shown in Figure 2A. From the trained latent space of the AI model, four high-affinity peptides MSK-1, MSK-2, MSK-3, and MSK-4 were selected for further investigation. Molecular docking analysis revealed that all four peptides demonstrate extensive binding coverage across the RBD, effectively blocking the viral interaction site. This steric blockade prevents the RBD from engaging with the human angiotensin-converting enzyme 2 (ACE2) receptor, thereby inhibiting viral attachment and cellular entry, as shown in Figure 2B.

#### 3.1.1. Peptide Toxicity and Allergenicity

To evaluate the toxicity and allergenicity of peptides, Allertop and ToxinPred, developed by Dimitrov et al., were employed [58,59]. These tools utilize machine-learning algorithms to classify proteins as allergens or non-allergens based on amino acid sequences and physicochemical properties. The peptide sequences were obtained from the VAE/WAE model and introduced into the servers. These tools offer valuable information on the potentially harmful effects and allergenic characteristics of peptides, as shown in Table 1.

#### 3.1.2. Physiochemical Properties of Predicted Antiviral Peptides

The physiochemical properties of antiviral peptides were predicted by utilizing two web servers, PROTOPARAM and APD3 [62]. The AVP prediction contains several properties, including charge, peptide mass, hydrophobicity value, PI (isoelectric point), half-life, instability index, and Boman index. To optimize the study and further enhance the focus, we prioritized antiviral peptides with maximum stability index and half-life. Finally, four antiviral peptides were selected out of fifteen based on their physiochemical properties such as isoelectric point, net charge, and other properties. The physicochemical properties of the four AVPs are given below in Table 2.

#### 3.1.3. Molecular Docking Analysis of MSK-1 and MSK-2

**Figure 3:**
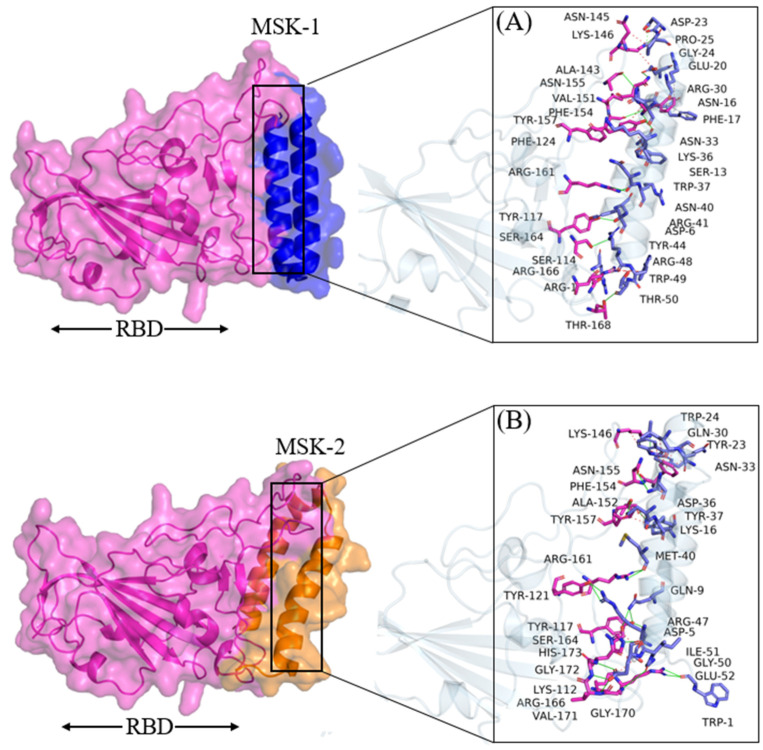
Docking analysis. (**A**) MSK-1 with RBD (**B**) MSK-2 with RBD Omicron variant.

HADDOCK predicted a docking score of −106.4 ± 4.3 for MSK-1 and 17 residues involved in the binding interaction. The residues involved in hydrogen bond interaction are Phe1, Gln8, Tyr11, Gln22, Trp35, and Arg47, as shown in Figure 3A. The electrostatic and Van der Waals energies were measured at −176.2 ± 9.6 and −74.5 ± 8.7, respectively, as shown in Table 3.

Moreover, the Phe154, Tyr114, and Arg46 reveal cation–π interactions and one salt bridge, respectively, which contributed to the high negative binding energy of these analogues to the protein. The cation–π interaction is due to molecular electron delocalization, reinforcing the importance of the increase in electron delocalization for the affinity [63,64]. Similarly, the docking score predicted for MSK-2 was −126.2 ± 5.6, 22. Residues Asp6, Ser13, Asn16, Glu20, Asp23, Asn33, Asn40, Tyr44, Arg48, and Thr50 form hydrogen bonds, while one residue, Asp23, forms a salt bridge interaction as shown in Figure 3B. The electrostatic and Van der Waals energies were calculated at −197.9 ± 30.2 and −88.1 ± 3.5, respectively, as shown in Table 2.

#### 3.1.4. Docking Analysis of MSK-3 and MSK-4

**Figure 4:**
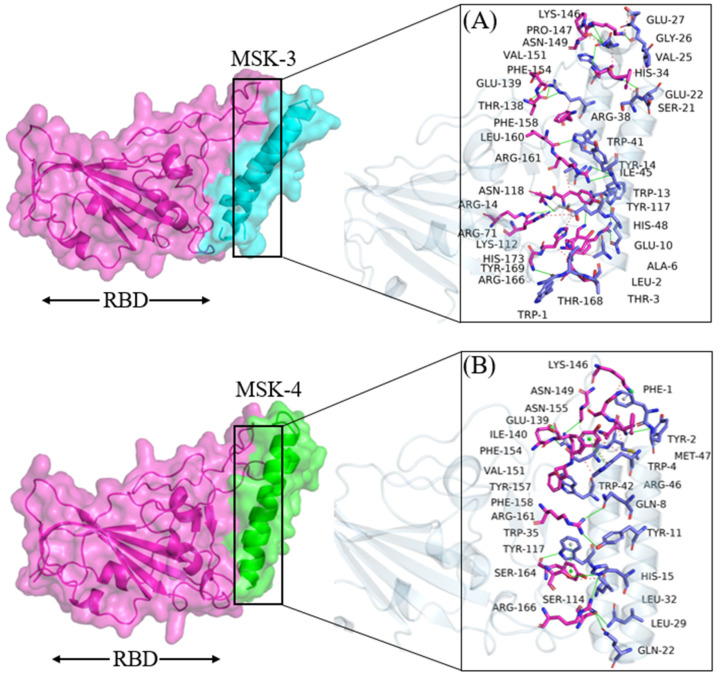
Molecular docking analysis of (**A**) MSK-3 and (**B**) MSK-4 with RBD.

In the same way, HADDOCK predicted a docking score of −125.7 ± 4.3 and −127.8 ± 4.3 for MSK-3 and MSK-4 with electrostatic and wander wall interactions of −277.3 ± 10.9, −283.3 ± 11.9 and −74.7 ± 4.9, −77.6 ± 3.6, respectively, as shown in Table 3, Furthermore, 29 residues involved in total binding interaction and the residues Trp1, Asp5, Gln9, Lys16, Asn33, Asp36, Met40, Arg47, Ile51, Glu52 form hydrogen bonds, while Asp45 and Tyr117 form salt bridge and cation–π interactions, respectively, as shown in Figure 4A. While in MSK-4, 17 residues are involved in binding interactions. Among them, Trp1, Gln9, Glu10, Gln17, Lys31, Arg38, Trp41, and His48 residues form hydrogen bond interactions, and three residues, Glu27, Arg38, Glu10, are involved in salt bridge interaction, as shown in Figure 4B. Furthermore, during molecular docking analysis, we observed that Glu10 and Arg38 in MSK-4 bonded with three residues on the surface of RBD, making the interaction stronger. The residues of the SARS-CoV-2 Omicron variant, which form hydrogen bonds and other interactions with antiviral peptides, are represented in Table 4.

#### 3.1.5. Root Mean Square Deviation (RMSD)

**Figure 5:**
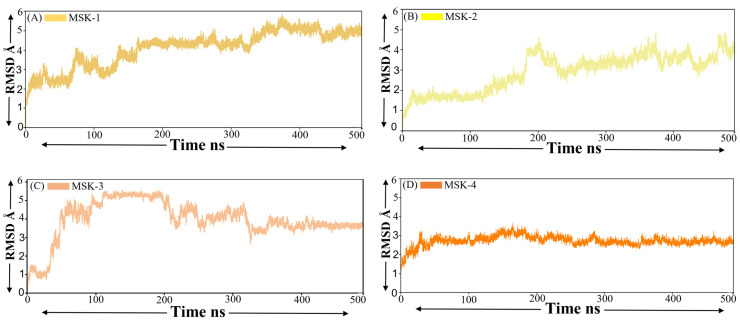
RMSD of four peptide complexes. (**A**) MSK-1 (**B**) MSK-2 (**C**) MSK-3, and (**D**) MSK-4. The *y* axis represents RMSD Å while the *x* axis represents time in ns.

The root mean square deviation RMSD is an essential parameter in molecular dynamics simulation, offering insight into the structural stability and conformational changes of molecules, particularly proteins or peptides, over time [65]. The RMSD of four peptides in complexes with the Omicron variant of SARS-CoV-2 provides a comprehensive insight into structural stability during the stability of MD simulation. The RMSD of MSK-1 complex starts with an initial rise from approximately 2 Å to around 4.1 Å up to 180 ns. Later, the RMSD revealed relatively moderate fluctuations between 4.1 Å and 4.6 Å for most of the simulation. Near the end of the simulation, a minor increase is observed with the RMSD approaching a value of 5.3 Å, indicating that the peptide experiences a degree of structural deviation; however, it maintains relative stability throughout the simulation period, as shown in Figure 4A. This suggests that MSK-1 maintains its structural integrity with minor conformational changes over time, suggesting a stable peptide structure. On the other hand, the RMSD of MSK-2 starts from 0.5 Å and increases within the first 200 ns, reaching a value up to 4.3 Å. Afterward, the RMSD fluctuates between 2.1 Å and 3.3 Å until 250 ns and then consistently shows stability between 250 and 400 ns and finally, again shows a minor fluctuation, as shown in Figure 5B. Similarly, in the case of MSK-3 complex, the RMSD displays some degree of fluctuation initially from 1 Å and gradually increases, reaching a value of around 4.9 Å by 100 ns, as shown in Figure 5C. However, MSK-2, and MSK-3 stabilize following the initial increase, with the RMSD value fluctuating between 3 Å and 4.9 Å for the remaining simulation. This pattern suggests that MSK-3 shows some conformational changes in the early stages, it reaches a relatively stable conformation midway through the simulation and maintains this conformation with minor fluctuations. Finally, the MSK-4 complex indicates that the RMSD values remained stable within a range of approximately 1.5 Å to 3.1 Å during the simulation, as shown in Figure 5D. Following an initial increase, the RMSD stabilizes within a range of approximately 2.5 Å, indicating that the complex reaches the equilibrium stage in the simulation. The absence of any significant sharp fluctuation suggests that the MSK-4-protein complex remains relatively stable during the simulation period. Finally, we confirmed from the root mean square deviation results that these four peptides maintain stable interactions, especially MSK-4, during the simulation period.

#### 3.1.6. Root Mean Square Fluctuation

**Figure 6:**
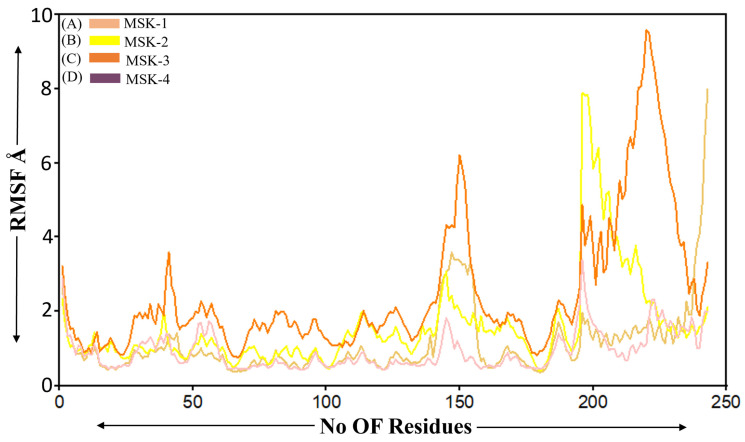
Root mean square fluctuation analysis of four complexes (**A**) MSK-1, (**B**) MSK-2, (**C**) MSK-3 and (**D**) MSK-4.

Root mean square deviation (RMSD) is frequently used in molecular dynamics simulations to investigate the flexibility of different regions inside biomolecular complexes [66]. We performed the RMSF analysis of four peptides in complex with the SARS-CoV-2 RBD Omicron variant. The overall trend among all peptides indicates that the RMSF values are comparatively low for the initial 150 residues, within an average fluctuation of approximately 2 Å, indicating a more stable region within the peptide structure. MSK-3 exhibited slightly more fluctuation initially, reaching a peak at approximately 3.6 Å, which indicates greater flexibility in specific regions compared to other peptides. Beyond the 200 residues, the MSK-2 and MSK-3 peptides demonstrated higher flexibility within a significant peak observed between residues 180–230 and 190–230, suggesting that these regions may relate to the loop region active site or disorder domain in the peptide structure, which is generally more flexible. The most significant fluctuation occurred in MSK-2 and MSK-3 with peaks at approximately 7.9 Å and 9.3, respectively, as shown in Figure 6. On the other hand, MSK-4 displayed smaller fluctuations throughout the simulation period, as shown in Figure 6. The RMSF analysis indicates that all four peptides in complex with the SARS-COVID-2 Omicron variant initially revealed structure similarity in terms of the stable region. Their flexibility varied significantly in the later residues, particularly for MSK-4, which demonstrated lower fluctuations over the simulation period suggesting that the MSK-4 plays a crucial role in their biological function or interaction.

#### 3.1.7. Radius of Gyration (ROG)

**Figure 7:**
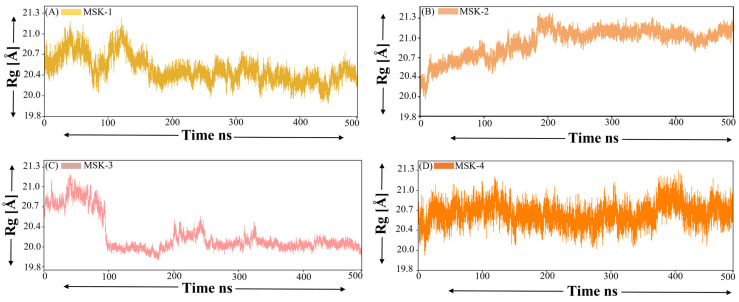
Radius of gyration of four complexes (**A**) Rg of MSK-1 complex, (**B**) MSK-2, (**C**) MSK-3, and (**D**) MSK-4.

The radius of gyration is a crucial parameter in molecular dynamics simulation, indicating the spatial distribution of atoms in a protein or peptide relative to its center of mass. ROG is widely used to examine the overall compactness and folding behavior of macromolecules during simulation. The Rg value of MSK-1 fluctuates between approximately 20.4 Å and 21.3 Å, indicating that it maintains a compact and stable structure during the simulation period, as shown in Figure 7A. However, a slight decrease in fluctuation is observed up to 190 ns with an Rg value around 20.3 Å. On the other hand, MSK-2 demonstrates variability in Rg fluctuation between 20.4 Å and 21.8 Å. The broader range shows that MSK-2 exhibits greater flexibility and undergoes more substantial conformational changes compared to MSK-1. Despite the Rg values being consistently greater than those of peptide 1, a visible fluctuation is observed, particularly around 0 to 200 ns and after, it shows stability with minor fluctuations during the whole simulation, as shown in Figure 7B. The fluctuation of Rg across a broader range may suggest that MSK-2 assumes both compact and extended conformations during the simulation. Similarly, MSK-3 initially increases up to 21.2 Å and then suddenly decreases until 100 ns. Afterward, MSK-3 showed stability for most of the simulation duration, as shown in Figure 7C. Finally, MSK-4 had the lowest Rg values among the four peptides, initially fluctuating around 20.2 Å and 20.9 Å up to 150 ns and then decreasing further, as shown in Figure 7D. The lower Rg value indicates that MSK-4 is more compact than the other peptides and sustained a more condensed structure during the simulation.

#### 3.1.8. Solvent-Accessible Surface Area

The solvent-accessible surface area SASA, mostly utilized in molecular dynamics simulation, is an essential parameter that represents the surface area of a biomolecule that is accessible to solvent, specifically water [67]. Changes in the SASA score offer insight into the folding, unfolding, or conformation changes of proteins or peptides, as they indicate the extent to which various regions of molecules are exposed to the surrounding solvent environment [68]. Solvent-accessible surface area analysis was performed for four peptides in complex with the SARS-CoV-2 Omicron variant. The nonpolar solvation energy (ΔGnonpolar) was computed utilizing the solvent-accessible surface area, SASA.

ΔGnonpolar = γ·SASA + β, ΔGnonpolar = γ·SASA + β

The SASA was calculated using the LCPO algorithm with a probe radius of 1.4 Å, and β = 0. All calculations were executed utilizing the amber module.

**Figure 8:**
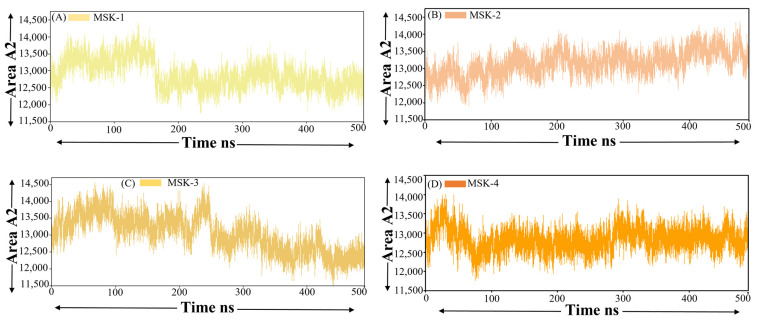
Solvent accessible surface area of four peptides in complex with Omicron RBD. (**A**) MSK-1 (**B**) MSK-2 (**C**) MSK-3, and (**D**) MSK-4.

The SASA value of MSK-1 fluctuated between 12,500 and Å^2^ 13,500 Å^2^ during the simulation period, as shown in Figure 8A. A minor decreasing trend in SASA was observed after 170 ns. This reduction may signify a progressive folding or compaction of the peptide structure leading to a less solvent-exposed surface area. Near the end of the simulation, the SASA exhibited a slight increase, suggesting a partial unfolding of the peptide. Despite these minor fluctuations, the overall SASA score of MSK-1 indicates a consistently stable solvent exposure exhibiting no substantial conformational changes during the simulation. In the same way, MSK-2 demonstrated a broader spectrum of SASA fluctuation with values ranging from around 12,500 Å^2^ to 13,700 Å^2^, as shown in Figure 8B. The SASA values of MSK-3 are comparatively higher, ranging from 12,000 Å^2^ to 14,000 Å^2^, as shown in Figure 8C. The SASA profile for MSK-3 exhibited significant variability during the simulation, characterized by both upward and downward fluctuations in solvent exposure. This indicates that MSK-3 undergoes dynamic conformational changes, fluctuating between folded and more expanded states. Finally, MSK-4 showed the lowest SASA values among the four peptide–protein complexes, fluctuating roughly between 12,200 Å^2^ and 13,800 Å^2^, as shown in Figure 8D. Initially MSK-4 revealed fluctuations up to 100 ns and after, maintained stability throughout the whole simulation period. The lower SASA scores indicate that MSK-4 retained a more compact structure during the simulation, exhibiting a smaller surface area exposed to solvent relative to other peptides. This compactness is specifically clear from the comparatively constant SASA score which exhibited smaller fluctuations compared to other peptides. A slight decrease in the SASA score was observed in the initial half of the simulation, followed by a progressive increase in the latter stage, which demonstrates that MSK-4 experienced a degree of compaction initially in the simulation, followed by minor structural modification. Finally, the SASA score revealed these peptides, particularly MSK-4, maintained stable interactions during the simulation period.

#### 3.1.9. Hydrogen Bond Analysis

**Figure 9:**
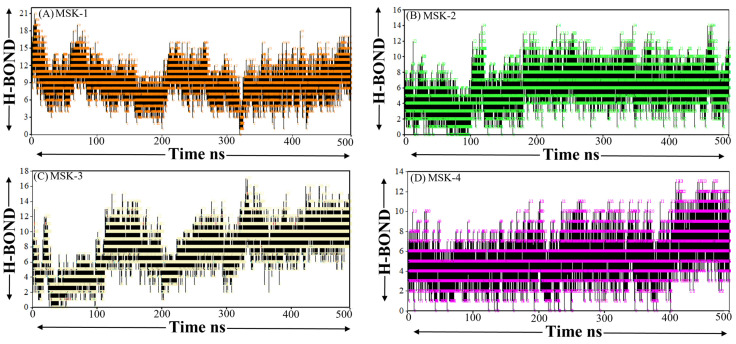
Hydrogen bond analysis (**A**) MSK-1 complex, (**B**) MSK-2, (**C**) MSK-3, and (**D**) MSK-4.

Hydrogen bonds are essential for maintaining the structural stability and conformation of peptides and proteins as they play a crucial role in secondary and tertiary structure elements. Evaluating the number and strength of hydrogen bonds can yield insights into the peptide’s flexibility, folding dynamics, and overall stability throughout the simulation. The number of hydrogen bonds in MSK-1 fluctuated between three and eighteen during the simulation, with a lower peak exceeding 2 H-bonds, as shown in Figure 9A. The overall pattern indicates a relatively dynamic hydrogen bonding characteristic. The fluctuation between 190 ns and 200 ns demonstrates significant changes, indicating that a period of structural rearrangement may be linked to modification in its conformational stability. Similarly, as compared to MSK-1, MSK-2 displays a higher and more stable hydrogen bond profile. The quantity of hydrogen bonds fluctuates initially between 0 and 10 and shows some peaks nearing the 14 H-bond, as shown in Figure 9B. This suggests that MSK-2 sustained a more stable internal hydrogen binding network during the simulation, indicating higher structure stability. The frequent fluctuation in the number of hydrogen bonds, especially at the midpoint of the simulation, may indicate a slight unfolding, but the overall consistency in hydrogen bond number indicates a well-formed and stable secondary structure. The higher number of hydrogen bonds, especially in the latter portion of the simulation, indicates that MSK-2 is likely to maintain a greater degree of its native conformation over time, resulting in a more stable and functionally significant structure. On the other hand, MSK-3 revealed a lower dynamic hydrogen bonding profile, showing more fluctuations between 0 and 10 up to 220 ns and later, showing stability during the last simulation period, as shown in Figure 9C. The higher peak of the hydrogen bond reached 12 and sometimes 14, indicating that MSK-3 establishes a strong hydrogen bonding network in a stable conformation. Finally, MSK-4 showed a more stable and consistent hydrogen bond profile with the number of hydrogen bonds fluctuating between two and ten over the simulation period, as shown in Figure 9D. Despite slight fluctuations, especially around 70 ns, the overall trend indicates that MSK-4 maintained stable hydrogen bond interaction through the simulation period.

#### 3.1.10. Dynamic Cross-Correlation Analysis (DCCM)

**Figure 10:**
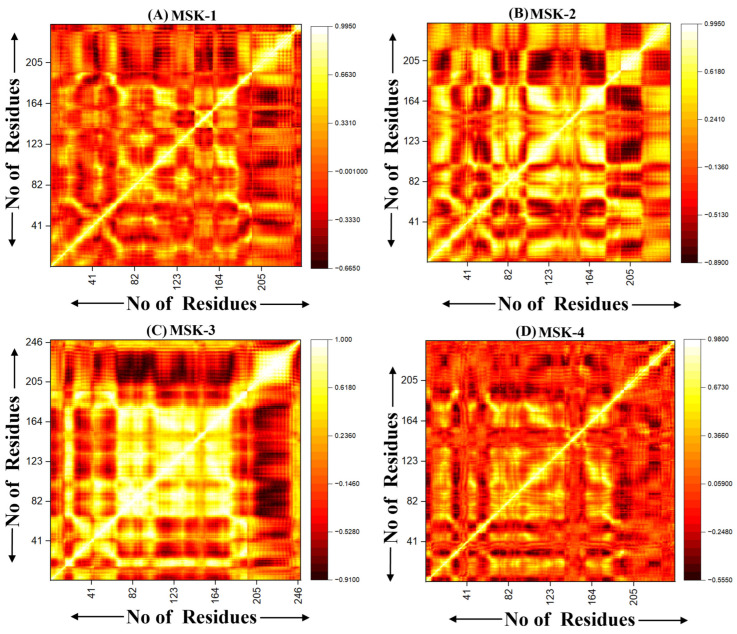
Dynamic cross-correlation of four peptides with RBD Omicron variant. (**A**) MSK-1 complex, (**B**) MSK-2 (**C**) MSK-3, and (**D**) MSK-4.

Dynamic cross-correlation motion DCCM analysis is an essential technique for understanding correlated motion within biomolecules, especially peptides and proteins [69]. It offers insight into the collective dynamics of molecular systems and highlights areas of correlated and anti-correlated motions that influence the dynamic stability and functional process of peptides [70]. We performed the Dynamic Cross-Correlation DCCM analysis for four peptide complexes. The DCCM analysis of MSK-1 displays regions of both correlated and anti-correlated moments. MSK-1 exhibits a distinct correlation pattern with the protuberant diagonal band, demonstrating a robust intra-residue correlation. Prominent clusters of positive correlation are observed along the diagonal, particularly in the lower and upper sections, suggesting that residues in these regions demonstrate coordinated motion, as shown in Figure 10A. These regions may align with secondary structures such as alpha helices or beta-sheets, where residues tend to move together. However, distinct areas of negative correlations off-diagonal indicate anti-correlated motions between different portions of peptides. In contrast, MSK-2 demonstrates a unique pattern of correlated and anti-correlated motion compared to MSK-1. There are significant regions of positive correlation, specifically in the center of the peptide–protein complex, indicating that greater portions of residue move in a coordinated manner. Particularly, the residues from 100 to 140 illustrate potential binding with the residues with the residues from 200 to 240, signifying efficient binding that plays a significant role in the stability of this peptide, as shown in Figure 10B. This may indicate a more compact structure where collective motion dominates over individual or localized fluctuations. Similarly, the DCCM analysis of MSK-3 exhibits a unique complex pattern with numerous strong off-diagonal correlations, specifically among residues 150 to 180 and 220 to 250. This indicates that despite being distant in sequence, these residues may experience rigorous movements, suggesting a specific functional role in the peptide’s mechanism of action. Several distinct areas display strong positive correlations, especially in the top left and bottom right quadrants of the graph, as shown in Figure 10C. These regions indicate that larger portions of the peptide participate in coordinated moments potentially enhancing the stability of overall structure. However, significant regions of anti-correlation exist, particularly between the N terminal and C terminal domain of the sequence indicating that these may move in opposite directions during the simulation. The DCCM matrices in the MSK-4 show an increase in inter-residue correlations, mainly between residues 50–80 and 180–210, indicate an increase in structural stability and binding due to due to cooperative movements among these regions. As compared to other peptides, the overall correlation pattern is less uniform, demonstrating complex dynamic behavior, which is crucial for its biological function, as shown in Figure 10D. As compared to MSK-3, MSK-4 exhibits a smaller number of extensive regions with pronounced anti-correlations, indicating that the overall complex structure is more rigid and undergoes less differential motion.

#### 3.1.11. Principal Components Analysis

**Figure 11:**
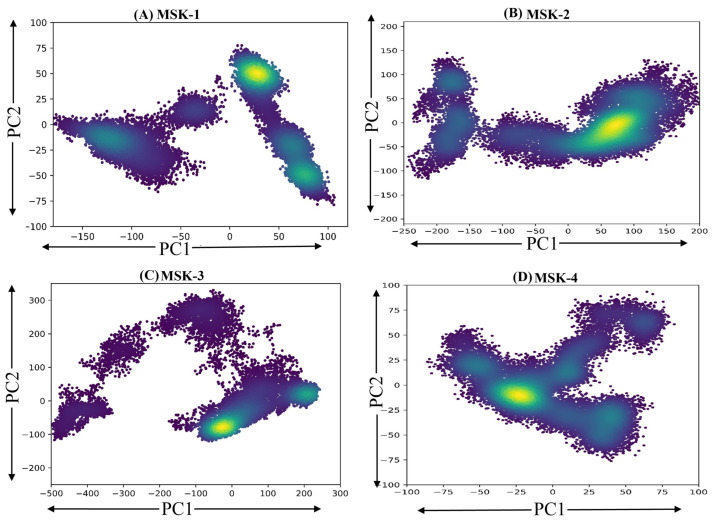
Principal components analysis of four peptides with RBD (**A**) MSK-1 (**B**) MSK-2, (**C**) MSK-3, and (**D**) MSK-4.

The principal component analysis (PCA) of four peptides in complex with the RBD of the SARS-CoV-2 Omicron variant was performed on the mean-centered data matrix X, labeled as MSK-1, MSK-2, MSK-3, and MSK-4, which provides insights into their structural stability and conformational dynamics (Figure 11). PCA reduces complex multidimensional data into two principal components, PC1 and PC2, elucidating the main source of variance in each peptide and enabling the comparative investigation of their stability through their scatter pattern in two-dimensional space. MSK-1 demonstrated a distinct clustering of points along the first (PC1), indicating substantial variance in its structural configurations. The distribution of data points suggests heterogeneity within this complex, with a concentration of points indicating a predominant structural conformation. In particular, the spread along the (PC2) revealed additional variability, suggesting that MSK-1 may adopt multiple conformations. On the other hand, MSK-2 displayed a more compact distribution of data points. The clustering along PC1 specifies a less diverse set of conformations compared to MSK-1. The comparatively uniform spread along PC2 advocates that the structural variability in MSK-2 is limited, potentially indicating a more stable interaction between the protein and peptide. Similarly, MSK-3 presents a complex landscape with a broad distribution of data points across both principal components. The presence of multiple clusters along PC1 suggests significant conformational diversity. This finding implies that the protein–peptide interactions in MSK-3 may be influenced by additional factors or interactions that promote structural variability. Finally, MSK-4 showed a unique distribution pattern, characterized by a pronounced separation of clusters along both PC1 and PC2. This indicates a distinct conformational landscape, with multiple conformations being represented within the dataset. The variation along PC1 suggests that MSK-4 may exhibit unique properties or interactions that differentiate it from the other complexes. In conclusion, the principal components analysis for the four peptides in complex with the RBD of the SARS-CoV-2 Omicron variant confirmed that these peptides show stability, especially MSK-4.

#### 3.1.12. MMGBSA Analysis

**Figure 12:**
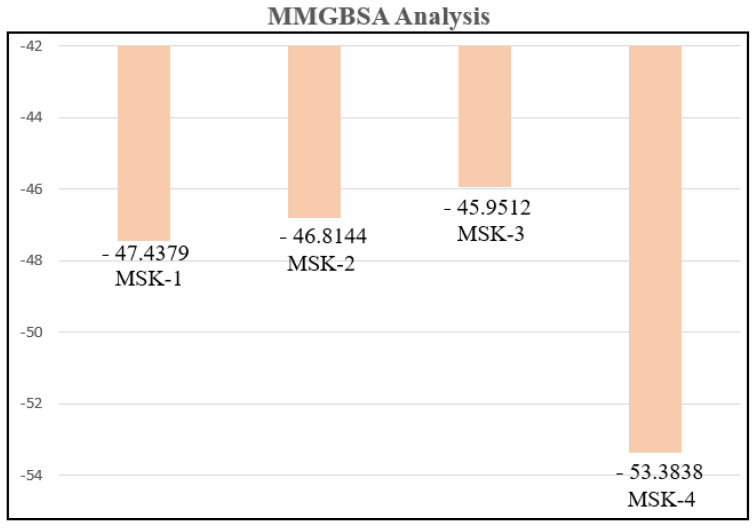
MMGBSA analysis of the top four peptides in complex with SARS-CoV-2.

The molecular mechanics generalized born surface area (MM/GBSA) approach is a commonly employed computational tool for calculating the binding free energy of biomolecular complexes, especially peptide–protein, ligand–protein, and protein–protein interactions [71]. MMGBSA integrates molecular mechanics (MM) energy calculation with solvation models, such as generalized born and surface area components, offering a comprehensive thermodynamics analysis of ligand or peptide interaction with the target [72]. This technique combines the advantage of molecular mechanics, which captures both bonded and non-bonded interactions, with the solvation model, allowing researchers to consider both polar and non-polar contributions to solvation energy [73]. The final 500 MD simulation frames were used to calculate the binding free energy of the four peptide complexes. The binding energies for MSK-1, MSK-2, MSK-3, and MSK-4 with the SARS-CoV-2 Omicron variant RBD were −47.4379, −46.8144, −45.9512, and −53.3838 kcal/mol, respectively. Van der Waals and electrostatic contributions are detailed in Table 5 and Figure 12. These results highlight MSK-4 as a particularly potent inhibitor of the SARS-CoV-2 Omicron variant.

## 4. Discussion

The spread of SARS-CoV-2 in late 2019 has resulted in a worldwide pandemic and caused significant global economic and social disruption [74]. With the passage of time, the SARS-CoV-2 virus mutated frequently, and several variants emerged, such as the Alpha, Beta, Gamma, Delta, and Omicron variants [75]. Omicron (B.1.1.529) was initially reported in South Africa on 24 November 2021 and has been identified by an extensive number of mutations in the spike protein, especially in the receptor binding domain, which performs an essential role in viral entry into the host cells [76]. These mutations have resulted in higher transmission and partial immune evasion, complicating the development of medicines and vaccines [77,78]. Currently, several subvariants of Omicron have emerged, such as BA.1, BA.2, BA.3, BA.4, and BA.5 [79]; therefore, we chose the Omicron variant for this study to control the future pandemic. SARS-CoV-2 persists in evolving due to the essential infidelity of RNA viruses, which produce random mutations alongside population immunity and a high rate of daily infection [80]. This mutation enables the virus’s potential to acquire resistance to current vaccines and therapeutics, particularly monoclonal antibodies and small-molecule drugs [81]. Therefore, there is an urgent need for the development of innovative antiviral peptide inhibitors that are capable of effectively targeting the virus. Thus, we implemented the variational autoencoder (VAE) and Wasserstein autoencoder (WAE) to develop innovative peptide inhibitors, specifically targeting the receptor binding domain of the SARS-CoV-2 Omicron variant. The structural changes in the spike protein of the Omicron variant pose a significant challenge to traditional therapeutic strategies, requiring an advanced computational approach for designing innovative peptide-based inhibitors. Both VAE and WAE were utilized because of their ability to effectively encode high-dimensional peptide sequences into a latent representation, facilitating the development of new and novel peptide sequences that demonstrate strong inhibitory activity. The VAE model facilitates sampling from the latent space, which intelligently generates various peptide sequences while ensuring that the new peptides maintain structural and functional relevance to known inhibitors. However, WAE focuses on minimizing the Wasserstein distance between encoded distributions, hence improving the quality and clarity of generated peptides by ensuring closer alignment between a latent space and the true peptide distribution. Comparative analysis of the newly generated peptide libraries suggests that both models produce effective inhibitors with substantial binding affinity to the receptor binding domain (RBD) of the SARS-CoV-2 Omicron variant. This finding highlights the significance of distributional regularization in producing high-quality peptide sequences, particularly in complex biological systems such as the SARS-CoV-2 Omicron variant. Furthermore, molecular dynamic simulation, binding affinity prediction, and MMGBSA analysis confirmed that four peptides generated by deep learning VAE and WAE models show stable interaction with crucial residues of the spike protein RBD of the Omicron variant, providing validation of their potential as effective inhibitors. Particularly, peptides produced by the WAE model exhibited more stability in secondary structures and a lower root mean square deviation during molecular dynamic simulation, therefore demonstrating the greater efficacy of WAE in developing structurally strong inhibitors. This research highlights the potential of deep learning generative models in addressing the issue raised by rapidly evolving viral pathogens and offers a framework for the development of peptide-based inhibitors for the treatment of viral proteins. Finally, the combination of the VAE and WAE models suggests an essential development in the computational design of antiviral peptides, offering an affordable and successful approach to generate potent inhibitors with potential applications against the RBD of the SARS-CoV-2 Omicron variant and other emerging viral threats. Further experimental work is necessary to provide a more comprehensive understanding of these peptide inhibitors against COVID-19 infection.

## 5. Conclusions

In this study, we have demonstrated that the new peptides constructed using artificial intelligence models, successfully block the receptor binding domain of SARS-CoV-2 and efficiently combat COVID-19 infection. Our data pave the way for the treatment of COVID-19 infections by the disruption of SARS-CoV-2-ACE2 interaction mechanisms and provide a structural basis to develop new medications against Omicron and future variants. Finally, the combined findings from the VAE/WAE models, MD simulations, and MMGBSA analysis indicate that all the peptides, especially MSK-4, exhibit significant potential inhibitory activity against SARS-CoV-2, with advantageous binding characteristics that suggest their efficiency in blocking the interaction between the SARS-CoV-2 RBD and the ACE2 protein. Further experimental work is necessary to validate our computational insights and gain a more comprehensive understanding of these peptide inhibitors against COVID-19 infection.

## Figures and Tables

**Table 1:** Toxicity and allergenicity of constructed peptides.

Name	Peptide Seq	Toxicity	Solubility	Allergenicity
MSK-1	FYNWLDKQHRYIFHHIFVHIRQDN SAVSLASLVKQTTNKFTWEARMD	Non-toxic	Goodwater solubility	Non-allergen
MSK-2	RPKQLDKQHNRASYWNFYHERQ DGPPNSYRLANLVKWTKNRQTYE ETRWT	Non-toxic	Goodwater solubility	Non-allergen
MSK-3 MSK-4	WLTLDARRQEEYWYRKQKAETS EYWVGEELQKENHADYRKMWN EAIYRHSGIEL WLTLDARRQEEYWYRKQKETSE YWVGEELQKENHADYRKMWNE AIYRHSG	Non-toxic Non-toxic	Goodwater solubility Goodwater solubility	Non-allergen Non-allergen
MSK-5	STIEE----SSLAS	Non-toxic	Goodwater solubility	Allergen
	GKGDFRI [60]	Non-toxic	Goodwater solubility	Allergen
	QAKTFLD [61]	Non-toxic	Goodwater solubility	Allergen

**Table 2:** Physiochemical properties of predicted antiviral peptides.

Peptide	Length	Pep Mass Dalton	Charge	Pi	Hydrophobicity (Wimley–White Whole-Residue)	Hydropathy Value	Boman Index (kcal/mol)
MSK-1	47	5793.57	+3	9.40	3.38	−0.55	2.16 kcal/mol
MSK-2	50	6308.95	+5.5	9.99	12.57	−1.87	3.97 kcal/mol
MSK-3	54	6835.55	−1.5	5.62	17.69	−1.42	3.2 kcal/mol
MSK-4	50	6409.03	−0.5	6.11	16.37	−1.67	3.55 kcal/mol

**Table 3:** HADDOCK predicted docking scores, cluster size, vdW energy, electrostatic energy, and Z-score for all new peptide complexes. VdW: Van der Waals, EE: electrostatic energy, BSA: buried surface area.

Parameter	MSK-1	MSK-2	MSK-3	MSK-4
HADDOCK score	−106.4 ± 4.3	−126.2 ± 5.6	−125.7 ± 4.3	−127.8 ± 4.3
Cluster size	17	29	28	26
RMSD	10.0 ± 0.4	11.6 ± 0.0	0.4 ± 0.2	0.8 ± 0.2
VdW energy	−74.5 ± 8.7	−88.1 ± 3.5	−74.7 ± 4.9	−77.6 ± 3.6
Electrostatic energy	−176.2 ± 9.6	−197.9 ± 30.2	−277.3 ± 10.9	−283.3 ± 11.9
Desolvation energy	−42.4 ± 3.7	−42.1 ± 2.5	−23.2 ± 2.6	−42.2 ± 2.9
Restraint’s violation of energy	457.6 ± 37.1	436.0 ± 25.3	467.2 ± 66.6	447.2 ± 65.6
Buried Surface Area	2111.3 ± 99.9	2569.3 ± 79.7	2260.1 ± 155.1	2150.1 ± 148.1
Z-score	−2.3	−1.8	−2.0	−2.4

**Table 4:** Interacting residues between SARS-CoV-2 and antiviral peptides during molecular dynamics simulation.

Peptides	Hydrogen Bond Interaction Residues	Other Interactions	Salt Bridge Interaction	π-Cation Interaction Residues
MSK-1	Tyr117,Arg166,Glu139,Asn149, Gly153,Arg161,ALA143, Thr168	Asn145, Lys146, Val151, Phe154, Tyr157, Tyr157, Phe124, Ser164, Ser114,	Arg46,	Phe154, Tyr114
MSK-2	Tyr1117,Ala143,Ala152, Phe154,Asn155,Arg161, Ser164, Arg166, Thr168	Lys146, Tyr157, Tyr121, His173, Gly172, Lys112, Val171	Asp23,	N/A
MSK-3	Lys112, Tyr117, Tyr121, Lys146, Ala152, Asn155, Tyr157, arg161, Ser162, Arg166, Glu172, His173	Pro147, Asn149, Val151, Phe154, Glu139, Thr138, Phe158, Leu160, Asn118, Arg114, Tyr169, Arg71	Asp45	Tyr117
MSK-4	Lys112, Glu139, Lys146, Pro147, Cyc148, Asn149, Phe154, Leu160, Arg161, Glu172, His173	Asn155, Ile140, Val151, Tyr157, Phe158, Trp35, Tyr117, Ser164, Arg166, Ser114	Glu27, Arg38, Glu10	N/A

**Table 5:** MM-GBSA binding free energy calculations, with all energies calculated in kcal/mol.

No.	Peptides	VDWAALS	EGB	EEL	ESURF	ΔTotal
2	MSK-1	−72.1669	−45.7232	95.7396	−9.2874	−47.4379
3	MSK-2	−62.0268	−86.7830	110.4271	−8.4317	−46.8144
4	MSK-3	−74.8832	383.2921	−344.3527	−10.0074	−45.9512
5	MSK-4	−59.7891	327.9998	−294.1442	−9.4503	−53.3838

## Data Availability

The datasets generated and analyzed in this study are available from the corresponding author upon reasonable request.
